# 

**Published:** 1886-10-23

**Authors:** 


### THE HOSPITAL

An Institution, Family, and Parochial Journal of
Hospitals, Asylums, & all Agencies for the Care of the Sick, Criticism & News.

### CONTENTS.

Poor-Law Infirmaries, Medical Education, and the People - - - 51
A Noble Profession: Nursing the Sick.—Half a Century Ago.—Later Developments.—Training of Nurses - 52
Discharged. Not where to Lay his Head. By George Fleming. Chapter V - - - 53
The Story of the Hospitals. By One of the Public—*continued* - - - 55
Words of Consolation.—The Divine Purpose - - 57
Poetry.—In a Hospital Ward - - - 57
Special Notices.—Conversazione of Hospital Supporters.—In and Out-Patients' Hospital Diary - - - 58
Notes and News.—Miscellaneous.—London School of Medicine for Women.—Large Legacies.—Durham County Hospital.—West Bromwich District Hospital.—The proposed Hospital for West Ham.—New Cottage Hospital at Abingdon.—"Sister Dora."—Wanted: Fruit and Linen for Hospitals.—Pictures in the Wards.—Mental Excitement: a Competition Item, etc., etc. - - 58
Annotations.—The Metropolitan Local Press.—Advertising Doctors and Secret Remedies.—A Just Complaint.—Hospitals and Dispensers - - - 61
Good Asylum Attendants for the Insane. Of Interest to All.—Published Facts.—Fifty Years Ago, and Since. The Ideal Attendant.—The Present Want - - 62
Till the Doctor Comes. II.—Diseases of Children.—Feverishness.—Teething.—The First Teeth.—Thrush.—Discharge from the Eyes.—Eczema of Head - - 64
Flowers, Ferns, and Ward Decorations - - - 65
Prize Competitions. Subjects and Awards - - - 65
The Hospital Charity Box. Prizes and Results - - 66
The Children's Corner. Puzzles, Answers, etc. - - 66
The In-Patients' Hospital Diary: General and Special Hospitals - - - 67

